# Parity and breast cancer risk among *BRCA1 *and *BRCA2 *mutation carriers

**DOI:** 10.1186/bcr1630

**Published:** 2006-12-22

**Authors:** Antonis C Antoniou, Andrew Shenton, Eamonn R Maher, Emma Watson, Emma Woodward, Fiona Lalloo, Douglas F Easton, D Gareth Evans

**Affiliations:** 1 CR-UK Genetic Epidemiology Unit, Strangeways Research Laboratory, Department of Public Health and Primary Care, Worts Causeway, University of Cambridge, Cambridge, CB1 8RN, UK; 2 Department of Clinical Genetics (SM2), St Mary's Hospital, Hathersage Road, Manchester, M13 0JH, UK; 3 Academic Unit of Medical Genetics and Regional Genetics Service, St Mary's Hospital, Manchester M13 0JH, UK; 4 Division of Medical Genetics, University of Birmingham School of Medicine, Birmingham, B15 2TT, UK

## Abstract

**Introduction:**

Increasing parity and age at first full-term pregnancy are established risk factors for breast cancer in the general population. However, their effects among *BRCA1 *and *BRCA2 *mutation carriers is still under debate. We used retrospective data on *BRCA1 *and *BRCA2 *mutation carriers from the UK to assess the effects of parity-related variables on breast cancer risk.

**Methods:**

The data set included 457 mutation carriers who developed breast cancer (cases) and 332 healthy mutation carriers (controls), ascertained through families seen in genetic clinics. Hazard ratios were estimated by using a weighted cohort approach.

**Results:**

Parous *BRCA1 *and *BRCA2 *mutation carriers were at a significantly lower risk of developing breast cancer (hazard ratio 0.54, 95% confidence interval 0.37 to 0.81; *p *= 0.002). The protective effect was observed only among carriers who were older than 40 years. Increasing age at first live birth was associated with an increased breast cancer risk among *BRCA2 *mutation carriers (*p *trend = 0.002) but not *BRCA1 *carriers. However, the analysis by age at first live birth was based on small numbers.

**Conclusion:**

The results suggest that the relative risks of breast cancer associated with parity among *BRCA1 *and *BRCA2 *mutation carriers may be similar to those in the general population and that reproductive history may be used to improve risk prediction in carriers.

## Introduction

Deleterious mutations in the *BRCA1 *and *BRCA2 *genes are associated with high risks of breast and ovarian cancer [1]. However, there is evidence that these risks are modified by both genetic and environmental factors [1-4]. Breast cancer risk in the general population is closely related to reproductive history, and reproductive factors are therefore strong candidates for modifiers of breast cancer risk in *BRCA1 *and *BRCA2 *mutation carriers. In particular, increasing parity has been shown to be protective for breast cancer in the general population in many studies [5-7], but its effect among *BRCA1 *and *BRCA2 *mutation carriers is still under debate [8-14]. In this report we have used data from 810 *BRCA1 *and *BRCA2 *mutation carriers from the UK to assess the effect of parity on breast cancer risk.

## Materials and methods

Families with breast and/or ovarian cancer have been tested for *BRCA1/2 *mutations since 1996 in the overlapping regions of North-West England and the West Midlands, covering about 10 million people. Women attending the specialist genetic clinics in these two regions with a family history of breast or ovarian cancer have a detailed three-generation family tree elicited. If a pathogenic *BRCA1/2 *mutation is identified, further attempts are made to ensure that all individuals relevant to discussions (those who could potentially carry any predisposing mutation) on risk are represented on the family tree. For the purposes of this analysis, pathogenic mutations include frameshift mutations, pathogenic splice variants, large rearrangements, or missense mutations classified as pathogenic by Breast Cancer Information Core [15]. All cases of breast or abdominal cancers are confirmed by means of hospital or pathology records from the Regional Cancer Registries (data available from 1960) or from death certification. Once a family-specific pathogenic *BRCA1/2 *mutation is identified, predictive testing is offered to all blood relatives. Where possible all affected women with breast or ovarian cancer are tested to establish the true extent of *BRCA1/2 *involvement in the family. In many instances this is done by obtaining paraffin-embedded tumour block material from deceased relatives. In many large families it is possible to establish 'obligate' gene carriers by testing for the same mutation in different branches of the family, thereby establishing that intervening relatives carry the same mutation.

All female *BRCA1/2 *mutation carriers identified by the regional genetics services were eligible for this study; their details and those of all tested relatives and first-degree untested female relatives were entered in a Filemaker Pro 5 database. The initial individual in which a mutation was identified was designated the 'index' case, with all other individuals being classified as to their position in the pedigree compared with a proven mutation carrier. Dates of births for the first and last completed third-trimester pregnancies, before breast cancer, or last follow-up were entered in the database for each of these women. The exception was mothers of a mutation carrier when it was clear that the mutation was paternally inherited. This study was approved by the Central Manchester Local research ethics committee, and participants consented to the Genetics Register research.

A total of 476 *BRCA1 *and 334 *BRCA2 *mutation carriers were used in a retrospective analysis of parity. Twenty-one parous carriers with a missing date at first live birth or for whom the age at first live birth could not be estimated from the available data were excluded from the analyses. The remaining individuals were censored (for instance, follow up was completed) at the age of breast cancer diagnosis (457), the age at ovarian cancer diagnosis (138), the age at mastectomy (37), the age at death (31) or the age at interview or last follow-up (126), whichever occurred first. Only carriers who were censored at breast cancer diagnosis were assumed to be affected. Among the 789 *BRCA1 *and *BRCA2 *mutation carriers included in the analyses, 647 were tested carriers and 142 were classified as obligate carriers by virtue of their position in the pedigree. The carriers originated from 392 distinct families. In all analyses we considered the number of full-term pregnancies occurring before the censoring age.

The effect of parity on breast cancer risk among *BRCA1 *and *BRCA2 *mutation carriers was assessed with the use of time-dependent Cox regression, and the effects are expressed as hazard ratios (HRs). According to the sampling design, affected mutation carriers may be preferentially sampled. In standard Cox regression the HR is estimated by comparing the distribution of the risk factor among affected individuals with the distribution among unaffected individuals at a particular time point. In the present study design, affected individuals are compared with unaffected carriers who were selected on the basis of their future disease status. However, if a risk factor was associated with breast cancer, then the risk factor would be over-represented among these unaffected carriers (because they would develop breast cancer in the future) and the HR under standard Cox regression would be biased towards 1 [16]. To correct for this potential bias we used a weighted cohort approach to analyse the effect of parity [16]. Under this method, individuals are weighted such that the observed breast cancer incidence rates in the study sample are consistent with established estimates of breast cancer risk among *BRCA1 *and *BRCA2 *mutation carriers. It has been shown that this approach gives estimates that are close to unbiased [16]. Weights were computed separately for *BRCA1 *and *BRCA2 *mutation carriers by using the breast cancer incidence rate estimates reported in the meta-analysis of Antoniou and colleagues [1].

*BRCA1 *and *BRCA2 *mutation carriers were considered in both combined and separate analyses. All analyses were stratified by the year of birth (before 1940, 1940 to 1949, 1950 to 1959, 1960 and later) and adjusted for oophorectomy. The joint analysis was also stratified by gene. We allowed for the fact that several individuals may come from the same family by using robust variance estimation [17,18]. For direct comparison we present our results in a similar format to that in the report of Andrieu and colleagues [19]. Because only age at first and age at last birth were recorded for carriers with more than two live births, intermediate births were assumed to occur at equally spaced intervals. Analyses were performed with STATA version 9 statistical software (Stata Corporation, College Station, TX, USA).

## Results

Table 1 shows a summary of the characteristics for the affected and unaffected carriers used in the analysis. The mean age at diagnosis for the affected individuals was lower than the censoring age for the unaffected carriers (43.4 and 47.3 years, respectively). The mean age at censoring was similar for *BRCA1 *and *BRCA2 *mutation carriers (44.7 and 45.4 years, respectively).

The results of the parity analyses using weighted Cox regression are shown in Table 2. Parous mutation carriers had a significantly lower risk of developing breast cancer (HR = 0.54, 95% confidence interval (CI) 0.37 to 0.81; *p *= 0.002). Having at least one live birth was protective in both *BRCA1 *and *BRCA2 *mutation carriers, and the HR did not differ significantly by gene, although the effect was statistically significant only among *BRCA1 *mutation carriers (*p *= 0.006). When the data were analysed by attained age, parity was found to be protective only among carriers over the age of 40 years (HR = 0.44, 95% CI 0.23 to 0.84; *p *= 0.013; *p *= 0.048 for the difference in HR between 40 years or less and more than 40 years). For mutation carriers under the age of 40 years, the HR was estimated to be 1.01 (95% CI 0.58 to 1.76).

There was evidence that increasing number of live births was associated with a decreasing risk of breast cancer in *BRCA1 *and *BRCA2 *mutation carriers combined. Each additional live birth was found to reduce breast cancer risk by 10% (HR = 0.90, 95% CI 0.80 to 1.00; *p *trend = 0.058). The reduction in risk with each additional birth was similar in *BRCA1 *and *BRCA2 *mutation carriers. However, two or more births conferred significantly lower breast cancer risk only among *BRCA1 *mutation carriers. There was some suggestion that increasing age at first live birth increased the risk of breast cancer among mutation carriers, but this was not statistically significant (for age 30 years or more at first live birth, HR = 1.30, 95% CI 0.74 to 2.29; *p *trend = 0.38). The effect seemed to be stronger in *BRCA2 *mutation carriers, particularly for age 30 years and over (HR = 4.77, 95% CI 2.08 to 10.94; *p *trend = 0.002), but the latter estimate was based on only 32 cases.

## Discussion

It is well established that increasing parity and early age at first birth are associated with a lower risk of developing breast cancer in the general population. There is evidence that the protective effect of parity may be restricted to women who are older than 40 years old [7,20-22]. Our results suggest that parity may have a similar effect on breast cancer risk among *BRCA1 *and *BRCA2 *mutation carriers. We found evidence that parous mutation carriers are associated with a lower risk of developing breast cancer and that the protective effect may be limited to carriers above the age of 40 years. This effect was observed only among *BRCA1 *mutation carriers, but there were no statistically significant differences in the estimates between *BRCA1 *and *BRCA2 *mutation carriers. There was no suggestion that parity might increase the risk of developing breast cancer in mutation carriers under the age of 40 years. There was evidence that all levels of parity are associated with a reduced risk of breast cancer among mutation carriers, but this was statistically significant only for carriers with two or more live births. There was also some suggestion of an increased risk associated with first birth after the age of 30 years, again consistent with the effect seen in the general population. However, this effect seemed to be restricted to *BRCA2 *mutation carriers. This difference might suggest differences in the development of breast cancer among *BRCA1 *and *BRCA2 *mutation carriers. Most breast cancer in *BRCA1 *mutation carriers arises in so-called 'basal' cells that stain positive for certain cytokeratins [23]. It might be that such cells are less responsive to the differentiation induced by pregnancy. However, it should be noted that the gene-specific effects of age at first birth were not a specific hypothesis in this study, and this difference may be attributable to chance.

Several studies, using a variety of designs, have investigated the effect of parity on breast cancer risk among *BRCA1 *and *BRCA2 *mutation carriers, but the results have not been consistent. Some studies have not found any significant association with parity [10,13,14], whereas others have reported that parity may increase the risk of breast cancer in mutation carriers [9,12]. Among these authors, Jernstrom and colleagues [12] found an increase in risk of breast cancer among carriers under the age of 40 years. However, this association was not present in a more recent, larger study by the same group [9], which is in line with our findings. In this latter report they found evidence of increasing risk with increasing parity among *BRCA2 *mutation carriers and that *BRCA1 *mutation carriers with four of more children were at a significantly lower risk of developing breast cancer than nulliparous carriers [9]. In contrast, we found evidence that increasing parity was protective among both *BRCA1 *and *BRCA2 *mutation carriers, and that *BRCA1 *mutation carriers with two or more offspring had a significantly lower breast cancer risk.

Our results are more in line with a recently published study by Andrieu and colleagues [19]. The estimates for the effects of parity from both studies are in the same direction and in some cases of similar magnitude. Andrieu and colleagues found that *BRCA1 *mutation carriers who had their first full-term pregnancy after the age of 30 years had a significantly lower risk of developing breast cancer. We did not find a significant evidence for this in the present study, but the confidence interval of our estimate includes the estimate of Andrieu and colleagues. As did Andrieu and colleagues [19], we found a significant association for breast cancer risk and pregnancy at older ages among *BRCA2 *mutation carriers. One hundred and forty-six carriers used in the present analysis were also used in the analysis by Andrieu and colleagues. We repeated our analysis by excluding those records. The results were virtually the same as those already presented, with similar conclusions (results not shown). The data overlap alone therefore does not explain the agreement between the two studies. Both the present analysis and that by Andrieu and colleagues [19] were performed with a weighted Cox regression approach, which may further contribute to the consistency between the studies.

As described above, our analyses also included obligate carriers. Since many of these obligate carriers are mothers of known carriers and are more likely to be affected, this may introduce some bias because they are by definition parous. We therefore repeated all the above analyses, excluding all obligate carriers (142 subjects). The estimated HRs were very similar to those obtained with the full data set (data not shown). Thus, there was no evidence that the inclusion of obligate carriers biased the results.

A major issue in the analysis of parity in *BRCA1 *and *BRCA2 *mutation carriers is that the decision to opt for genetic testing may itself be related to parity, because parous women may be more concerned to be tested. If the size of this selection bias differs between affected and unaffected carriers, this will bias the estimated risk associated with parity. This is an issue in all retrospective studies based on families opting for genetic testing, from which most of the relevant data can be obtained. We have attempted to reduce this possible bias by including information on reproductive history on all known carriers, including those who are obligate carriers or who have died from a relevant cancer. However, the possibility of selection bias cannot be ruled out. Population-based or prospective studies could avoid such biases, but the former are difficult to conduct and the latter will take many years to complete.

## Conclusion

Our results indicate that the relative risk of breast cancer associated with parity is similar to that in the general population. However, further larger studies are required to clarify the effect of age at first live birth on breast cancer risk among mutation carriers. Because *BRCA1 *and *BRCA2 *mutation carriers are susceptible to a much higher risk of breast cancer than non-carriers, the corresponding absolute differences in risk between parous and nulliparous carriers may be substantial. For illustration purposes consider an unaffected *BRCA1 *mutation carrier who is 40 years old. On the basis of the *BRCA1 *breast cancer incidence rates estimated by Antoniou and colleagues [1], the risk of developing breast cancer by the age of 70 years is 60%. Assuming that the estimated HR of 0.54 associated with parity applies to *BRCA1 *mutation carriers and using the same method as described by Mitchell and colleagues [24], the breast risk by 70 years of age for this mutation carrier is estimated to be 40% if she is parous and 61% if she is nulliparous. Thus, risk estimates used in genetic counselling could be improved by incorporating reproductive history, in a similar manner to that recently demonstrated for mammographic density [24]. However, given the inconsistencies between studies and the uncertainties in the age-specific risks, further large and preferably prospective studies will be required to provide more reliable absolute risk estimates.

## Abbreviations

CI = confidence interval; HR = hazard ratio.

## Competing interests

The authors declare that they have no competing interests.

## Authors' contributions

DGE was responsible for the conception and design of the study. DGE, FL, ERM and E Woodward provided the study patients. DGE, AS and E Watson collected and assembled the data. ACA, DFE and DGE performed the data analysis and interpretation. ACA, DFE and DGE wrote the manuscript. ACA, AS, ERM, E Woodward, FL, DFE and DGE read and approved the final manuscript.

## Figures and Tables

**Table 1:** Summary characteristics for affected and unaffected carriers

Parameter	Total	Affected		Unaffected	
		*BRCA1*	*BRCA2*	*BRCA1*	*BRCA2*
Number of carriers, *n*	789	248	209	218	114
Age at censoring, years (mean ± SD)	45.0 ± 11.3	42.9 ± 9.6	43.9 ± 9.3	46.8 ± 13.0	48.3 ± 13.0
Year of birth, *n *(%)					
Before 1940	247	78 (31.5)	64 (30.6)	78 (35.8)	27 (23.7)
1940 to 1949	185	69 (27.8)	61 (29.2)	40 (18.3)	15 (13.2)
1950 to 1959	169	58 (23.4)	48 (23.0)	34 (15.6)	29 (25.4)
1960 and after	188	43 (17.3)	36 (17.2)	66 (30.3)	43 (37.7)
Censoring age, *n *(%)					
<55 years	670	226 (91.1)	185 (88.5)	172 (78.9)	87 (76.3)
≥55 years	119	22 (8.9)	24 (11.5)	46 (21.1)	27 (23.7)
Oophorectomy before censoring age, *n*	45	5	7	25	8

**Table 2:** Results from weighted Cox regression analysis

Parameter	All				*BRCA1 *carriers				*BRCA2 *carriers			
	UN	BC	HR	95% CI	UN	BC	HR	95% CI	UN	BC	HR	95% CI
Parity												
Nulliparous	37	65	1.00		25	37	1.00		12	28	1.00	
Parous	295	392	0.54	0.37–0.81	193	211	0.53	0.34–0.83	102	181	0.58	0.27–1.24
Parity by attained age												
≤40 years												
Nulliparous	28	44	1.00		21	24	1.00		7	20	1.00	
Parous^a^	76	127	1.01	0.58–1.76	50	76	1.17	0.55–2.52	26	51	0.72	0.42–1.24
>40 years												
Nulliparous	9	21	1.00		4	13	1.00		5	8	1.00	
Parous	219	265	0.44	0.23–0.84	143	135	0.34	0.16–0.70	76	130	1.21	0.37–3.92
Number of live births^b^												
Nulliparous	37	65	1.00		25	37	1.00		12	28	1.00	
1	46	57	0.62	0.37–1.02	27	34	0.62	0.34–1.11	19	23	0.58	0.22–1.53
2	109	167	0.57	0.38–0.86	73	80	0.54	0.33–0.87	36	87	0.68	0.31–1.47
3	66	79	0.64	0.34–0.86	42	50	0.56	0.33–0.97	24	29	0.44	0.19–1.04
4+	56	82	0.55	0.34–0.91	42	44	0.51	0.29–0.89	14	38	0.71	0.27–1.88
Per live birth			0.90	0.80–1.00			0.89	0.78–1.01			0.93	0.75–1.14
Age at first live birth												
<20 years	61	55	1.00		38	35	1.00		23	20	1.00	
20–24 years	132	179	1.16	0.76–1.78	85	92	1.08	0.65–1.78	47	87	1.74	0.87–3.49
25–29 years	106	108	1.13	0.72–1.79	73	66	1.08	0.64–1.52	33	42	1.48	0.68–3.22
≥ 30 years	39	50	1.30	0.74–2.29	25	18	0.83	0.42–1.64	14	32	4.77	2.08–10.94
Nulliparous	37	65	2.09	1.26–3.45	25	37	1.96	1.10–3.52	12	28	2.85	1.13–7.15

UN, unaffected carriers; BC, breast cancer; HR, hazard ratio; CI, confidence interval. ^a^Analyses not adjusted for oophorectomy (all others adjusted). Overall analysis stratified by year of birth and gene. ^b^Assuming that births between first and last birth were equally spaced; 25 carriers (7 affected, 18 unaffected) with missing age at last birth were assumed to be nulliparous until age at first birth and were then considered as a separate category (hazard ratios not shown).
